# ZBTB7A, a miR-144-3p targeted gene, accelerates bladder cancer progression via downregulating HIC1 expression

**DOI:** 10.1186/s12935-022-02596-w

**Published:** 2022-05-02

**Authors:** Junqiang Liu, Zhiyuan Chou, Chun Li, Kai Huang, Xuejian Wang, Xiunan Li, Chuanchun Han, Abdullah Al-Danakh, Xiaodong Li, Xishuang Song

**Affiliations:** 1 Department of Urology of First Affiliated Hospital, Institute of Cancer Stem Cell, Dalian Medical University, Dalian, China; 2 Central Laboratory, Affiliated Zhongshan Hospital of Dalian University, Dalian, China

**Keywords:** ZBTB7A, HIC1, miR-144-3p, Bladder cancer

## Abstract

**Background:**

Zinc finger and BTB domain-containing 7A (ZBTB7A) is a member of the POK family of transcription factors that plays an oncogenic or tumor-suppressive role in different cancers depending on the type and genetic context of cancer. However, the function and molecular mechanism of ZBTB7A in bladder cancer (BC) remain elusive.

**Methods:**

The role of ZBTB7A in bladder cancer was detected by colony formation, transwell, and tumor formation assays. The expression levels of ZBTB7A, HIC1, and miR-144-3p were analyzed by qRT-PCR and Western blot. Bioinformatics analysis and a dual-luciferase reporter assay were used to assess the effect of ZBTB7A on the promoter activity of HIC1.

**Results:**

The present study revealed that knockdown of ZBTB7A suppressed BC cell growth and migration, as indicated by an approximately 50% reduction in the number of colonies and an approximately 70% reduction in the number of migrated cells. Loss of ZBTB7A inhibited tumor growth in vivo, resulting in a 75% decrease in tumor volume and an 80% decrease in tumor weight. Further mechanistic studies revealed that ZBTB7A bound to the hypermethylated in cancer 1 (HIC1) promoter and downregulated HIC1 expression, accelerating the malignant behavior of BC. Increased expression of ZBTB7A in BC tissues was negatively corrected with the expression of HIC1. Moreover, ZBTB7A was a target of miR-144-3p, which decreased ZBTB7A expression in BC.

**Conclusion:**

Our data demonstrate that ZBTB7A, a targeted gene of miR-144-3p, promoted tumorigenesis of BC through downregulating HIC1 expression.

**Supplementary Information:**

The online version contains supplementary material available at 10.1186/s12935-022-02596-w.

## Background

Bladder cancer (BC) is the most common malignancy of the urinary tract, with approximately 550,000 new cases annually [1, 2]. BC has been classified into two major subtypes, non-muscle-invasive BC (NMIBC) and muscle-invasive BC (MIBC) (75 and 25%) respectively [3, 4]. Despites Significant advancements in antitumor therapy through chemotherapy, surgery, and immunotherapy particularly immune checkpoint inhibitors [2], the 5 year survival rate for MIBC remains unsatisfactory. Thus, the identification of novel therapeutic targets and the development of individualized treatment strategies for BC are indispensable.

The zinc finger and BTB domain-containing 7A (ZBTB7A), also referred to as Pokemon (POK), LRF (lymphoma-related factor), or FBI-1 (factor binding IST protein 1), is a transcription factor that is a member of the POK family. ZBTB7A is a transcriptional regulator that binds with DNA via its zinc-finger domains [5, 6]. According to previous research, ZBTB7A may act as an oncogene or tumor suppressor in a variety of malignancies, depending on the type and genetic context of cancer. ZBTB7A was overexpressed in breast cancer tissue and promoted cell metastasis in vitro and in vivo via NF-κB induced epithelial-mesenchymal transition [7, 8]. Similarly, ZBTB7A was reported to be overexpressed in colorectal cancer tissues and was linked to a poor prognosis [9, 10]. In osteosarcoma, ZBTB7A has been shown to increase chemoresistance and protect osteosarcoma cells from ER stress-induced apoptosis [11, 12].

On the other hand, ZBTB7A has been found to decrease cell progression and metastasis in melanoma and prostate cancer patients [13–15]. Although ZBTB7A has been shown to act as an oncogenic or tumor suppressor protein in a variety of malignancies, its role and regulation mechanism in bladder cancer are poorly understood. The present study established that ZBTB7A levels were increased in BC tissues, promoting BC cell proliferation and migration via inhibiting hypermethylated in cancer 1 (HIC1) expression transcriptionally. Moreover, it was discovered that miR-144-3p was a target of ZBTB7A, resulting in a decrease in ZBTB7A expression in BC.

## Methods

### Cell culture and reagents

Two human urinary bladder cancer cell lines (T24 and UM-UC-3) that isolated from high-grade and late-stage tumors were used as MIBC models in this study [16]. T24 and UM-UC-3 were cultured in Dulbecco's Modified Eagle Medium (GIBCO-Invitrogen) containing 10% fetal bovine serum (ExCell Bio).The following antibodies were used in our study: Actin (Santa Cruz Biotechnology, sc-1616,1:1000), ZBTB7A (Santa Cruz Biotechnology, SC-33683, 1:1000), and HIC1 (Proteintech, 24949-1-AP, 1:1000), Mouse anti-Armenian hamster IgG-HRP (Santa Cruz Biotechnology, sc-2789, 1:2000) for ZBTB7A, HRP-conjugated Affinipure Goat Anti-Rabbit IgG(H + L) **(**Proteintech, SA00001-2, 1:10000**)** for HIC1**,** HRP-conjugated Affinipure Goat Anti-Mouse IgG(H + L) **(**Proteintech, SA00001-1, 1:10000) for Actin. The primary antibodies were incubated for two hours at room temperature, while the secondary antibodies were incubated for one hour.

### Cell migration and colony formation assays

In the colony formation assay, BC cells with the changed genes were diluted with single-cell suspension and we seeded 1000 or 2000 BC cells in each of the 6-well plates and incubated with 5% CO_2_ at 37 °C for one week. The colonies were then stained with 0.04% crystal violet in 2% ethanol and counted.

Bladder cancer cell migration assay was conducted and 1000 or 2000 BC cells were seeded in a 24-well Transwell plate with 8 mm polyethylene terephthalate membrane filters (Corning). Cells were allowed to migrate for 24 h at 37 °C in a humidification chamber containing 5% CO_2_. After incubation, the filter was removed and fixed with 4% formaldehyde for 15 min, followed by staining with 0.1% crystal violet for 20 min, and cells were counted.

### Sphere formation assay

Spheres were enriched from T24 and UM-UC-3 cells with or without ZBTB7A knockdown by culturing 1000 cells/mL in serum-free DMEM-F12 medium (Gibco) supplemented with B27 (1:50, Invitrogen) and 20 ng/mL EGF and bFGF. Nontreated tissue culture flasks were used to reduce cell adherence and support growth as undifferentiated tumor spheres. After two weeks of culture, the number of spheres with a diameter greater than 100 mm in each well was counted.

### Lentivirus packaging and infection

RNA interference was performed as previously described [17, 18]. Briefly, to generate the lentiviral shRNA constructs against human ZBTB7A and HIC1, the target sequences were cloned into pLKO.1-TRC vector. The following sequences were used to target ZBTB7A: No. 1 5-CCACTGAGACACAAACCTATT-3 and No. 2 5-GAACGTGTACGAGATCGACTT-3 and HIC1: No. 1 5-GCTGTGCAAGAAACGCCTCAA-3 and No. 2 5- TGATATCAGCTTTGACCAAAG-3. The pLKO.1, pVSVG, pREV and pGAG vectors were co-transfected into HEK293T cells for 48 h, and cell culture media were collected. The full-length ZBTB7A sequence were cloned into the pCDH-puro vector. pCDH, pSPAX.2 and pMD.2G were co-transfected into HEK293T cells for 48 h, and cell culture media were collected. The viruses for ZBTB7A or HIC1 knockdown or for ZBTB7A overexpression were isolated from the media and were used to infect BC cells in the presence of polybrene and after 48 h from infection, the cells were selected by puromycin and the expression of ZBTB7A and HIC1 were detected by Western blotting.

### MicroRNA mimics and inhibitors

miRNA-144-3p mimics and inhibitors were synthesized by GenePharma Company (Shanghai, China). For each transfection in a 6-well plate, 100 nM miR-144-3p mimics, scramble or inhibitors were used. Transfection of BC cells was performed using Oligofectamine (Invitrogen) following the manufacturer’s instructions. After transfection, the BC cells were still cultured for 36 h and then used for the following assays. For the colony formation assays, the miR-144-3p mimics and scramble were transfected into BC cells for two times. After 3 days for the first transfection, we transfected the miR-144-3p mimics and scramble again. After 4 days for the second transfection, the colonies were then stained with 0.04% crystal violet in 2% ethanol and counted.

### RNA sequencing analysis

10^6^ BC cells with or without ZBTB7A knockdown were collected and the cells were dissected using Trizol Reagent (Life Technologies, Inc. Lot: 113606), and the samples were transported in dry ice. RNA extraction, library construction, sequencing and data analysis were performed by BioMarker (Beijing, China). In summary, 3.0 μg of total RNA per sample was used as input material. RNA integrity was assessed using the RNA Nano 6000 Assay Kit (Agilent) on the Agilent 2100 BioAnalyzer. The ribosomal RNA was removed by Ribo-Zero^™^ Gold Kit (Epicentre, USA), and sequencing libraries were generated using the rRNA-depleted RNA by NEB Next Ultra Directional RNA LibraryPrep Kit for Illumina (NEB, USA). TDEGseq package (v1.18.0) was used for assessing differential gene expression and gene set enrichment was performed using the MSigDB with specific gene set collections (Hallmark, Cancer modules, GO).

### Real-time RT-PCR assay

Total RNA was isolated using Trizol (Invitrogen), and approximately 1 μg of total RNA was used for cDNA synthesis using the PrimeScript TM RT reagent kit (Takara, RR047A) according to the manufacturer’s instructions. For the PCR reaction, the 200 ng cDNA and 10 μM primers were mixed with 5 µl TB Green master mix (Takara, RR820A) and added the ddH2O to 10 µl reaction system for one sample. The following primers were used: actin: F: 5-GACCTGACTGACTACCTCATGAAGAT-3 and R: 5-GTCACACTTCATGATGGAGTTGAAGG-3; HIC1: F: 5-CGACAAGAGCTACAAGGACC-3 and R: 5- CAGATGGTGCATGGGTAGG -3. The primers for mature miR-144-3p and U6 were purchased from Takara. Actin was used as the reference gene for HIC1 analysis and U6 was used as the reference gene for miR-144-3p analysis. The ΔΔCt method was used for the relative gene expression calculation.

### Promoter reporters and dual-luciferase assay

For the analysis of HIC1 promoter activity, the promoters of HIC1 were cloned into the pGL3-basic vector. Then 1 μg of total plasmids were transfected into BC cells with or without ZBTB7A overexpression or knockdown. After transfection for 24 h, luciferase activity was measured in a 1.5 ml Eppendorf tube using the Promega dual-luciferase reporter assay kit (Promega E1980) following the manufacturer’s protocol. The relative Renilla luciferase activity was normalized to the firefly luciferase activity.

To analyze ZBTB7A 3’UTR activity, the ZBTB7A 3’UTR were constructed into the psiCHECK2 vector. After that, 1 μg of total plasmids were transfected into BC cells together with miR-144-3p mimics or inhibitor. Following transfection for 24 h, luciferase activity was measured in a 1.5 ml Eppendorf tube using the Promega dual-luciferase reporter assay kit (Promega E1980) according to the manufacturer’s protocol. The relative firefly luciferase activity was normalized to the Renilla luciferase activity.

### Chromatin immunoprecipitation assay

Chromatin immunoprecipitation tests were performed using the Millipore ChIP kit (17-371RF) following the manufacturer’s instructions. The 10 μg ZBTB7A antibody (Santa Cruz Biotechnology, SC-33683X) was used. The following specific primers HIC1 promoter F: 5-GGATCTGGGCGCACCTCA-3 and R: 5- GTCTACGGGGAGTTCC-3 were used for RT-PCR for binding DNA fragments.

### Immunohistochemistry (IHC)

The BC tissue microarrays purchased from Shanghai Outdo Biotech Co. Ltd. (Shanghai, China), contained 30 pairs of cancer tumors together with matched adjacent normal tissue. IHC staining was performed for ZBTB7A (Santa Cruz Biotechnology, SC-33683, 1:200) and HIC1 (Proteintech, 24949-1-AP, 1:200) on the same paraffin-embedded tissue blocks used for clinical diagnosis. IHC was performed using the avidin–biotin complex method (Vector Laboratories), including heat-induced antigen-retrieval procedures.

The degrees of immunostaining were reviewed and scored by two independent pathologists. The proportion of the stained cells and the extent of staining were employed as evaluation criteria, with at least 1000 tumor cells evaluated in each case. While one score was assigned based on the percentage of positive cells, another was assigned based on the staining intensity, and the final score was calculated by multiplying the two previous scores.

### Flow cytometric analysis

Bladder cancer cells with or without ZBTB7A knockdown were harvested for apoptosis analysis. The samples were then processed for Annexin V and PI double-staining with an apoptosis assay kit (Yeasen, China) as per the manufacturer’s instructions, and flow cytometry was used to identify apoptosis in cells (BD Accuri C6).

### In vivo tumorigenesis

Animal experiments were performed according to the National Institute of Health Guide for the Care and Use of Laboratory Animals and approved by the Animal Research and Care Committee of Dalian Medical University. Male nude mice (4–6 weeks old, 18–20 g) were obtained from SPF Laboratory Animal Center of Dalian Medical University (Dalian, China) and were randomly divided into the indicated groups. T24 cells with or without ZBTB7A knockdown were subcutaneously injected into nude mice. After 7 days, the size of the tumor was measured using Vernier calipers every 2 days and converted into tumor volume (TV) according to the following formula:

$$ {\text{TV }}\left( {{\text{mm}}^{{3}} } \right)\, = \,\left( {{\text{a }} \times {\text{ b}}^{{2}} } \right)/{2}, $$

where a and b are the maximum and minimum diameters, respectively. All animals were killed 31 days after injection, and the transplanted tumors were removed and weighed.

### Statistical analysis

To establish the statistical significance of differences between separate groups, a two-tailed paired t-test, and a two-way ANOVA were performed, and error bars represent the standard deviation of the mean (SD). GraphPad Prism 7 was used for statistical analysis unless otherwise specified. The data are presented as mean ± SD and the level of significance was set at *p < 0.05, **p < 0.01 and ***p < 0.001.

## Results

### ZBTB7A promotes BC cell growth and migration in vitro and in vivo

**Fig. 1:**
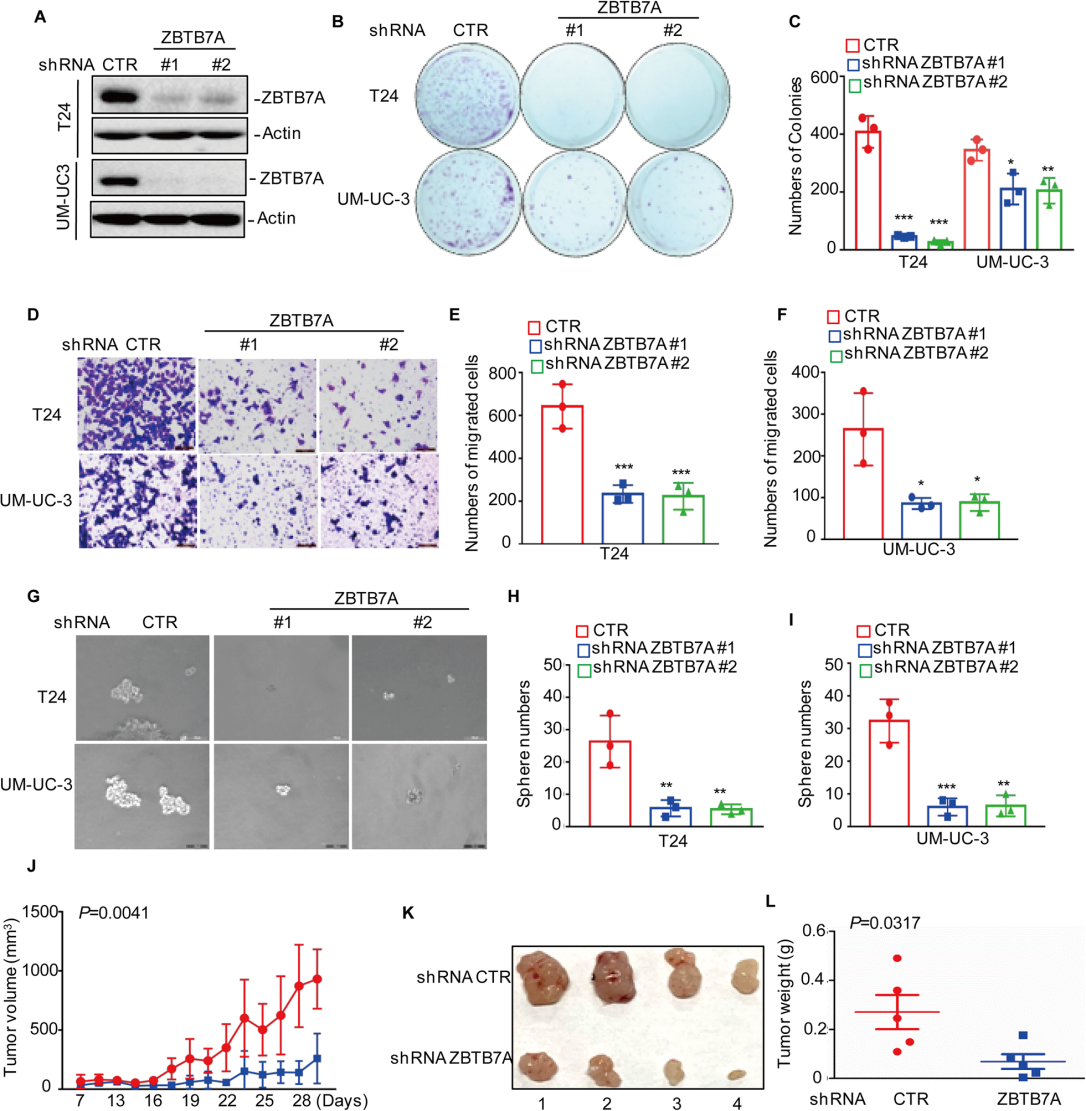
ZBTB7A promotes BC tumorigenesis. **A** ZBTB7A was knocked down in two BC cells (T24 and UM-UC-3) and the expression levels of ZBTB7A were detected using Western blotting. **B–F** Cell growth and migration were assessed by colony formation and transwell assays. **G–I** sphere-forming abilities of T24 and UM-UC-3 cells with or without ZBTB7A knockdown. **J–L**. Tumor-forming abilities of T24 cells with or without ZBTB7A knockdown were measured. The tumor volume and weight were assessed. All results represent three independent experiments and presented as the mean ± SD. *p < 0.05, **p < 0.01 and ***p < 0.001 compared with the control group

To evaluate the role of ZBTB7A in the development of human BC, ZBTB7A expression was knocked down in T24 and UM-UC-3 cells using two independent shRNAs. ZBTB7A expression levels were significantly downregulated in shRNA ZBTB7A groups compared to the control group (Fig. 1A). Following that, we used a colony formation test and flow cytometric analysis to examine the effect of ZBTB7A knockdown on cell proliferation and death. The results showed that inhibiting ZBTB7A expression significantly reduced the number of colonies in treated groups compared to controls, but had no effect on cell death (Fig. 1B, C and Additional file 1: Fig.S1A, B). Transwell experiments demonstrated that the knockdown of ZBTB7A significantly decreased the number of migrating cells (Fig. 1 D, F). To further elucidate ZBTB7A's oncogenic potential in BC, the effect of ZBTB7A knockdown on the capabilities of cancer stem cells revealed that ZBTB7A knockdown lowered BC cells' sphere-forming efficiency (Fig. 1G–I).

The effect of ZBTB7A on tumor growth in vivo was then investigated using T24 cells because ZBTB7A inhibited colony formation more significantly in T24 cells than in UM-UC-3 cells as shown previously. The T24 cells with or without ZBTB7A knockdown were implanted in nude mice and we found that, compared with control cells, loss of ZBTB7A significantly suppressed the growth of T24 xenograft tumors, as indicated by tumor weight and size reduction (Fig. 1J–L), also the downregulation of ZBTB7A was confirmed by Western blotting in tumor xenografts (Additional file 1: Fig S1C). In summary, our result demonstrated the oncogenic role of ZBTB7A in BC.

### ZBTB7A transcriptionally suppresses HIC1 expression in BC

**Fig. 2:**
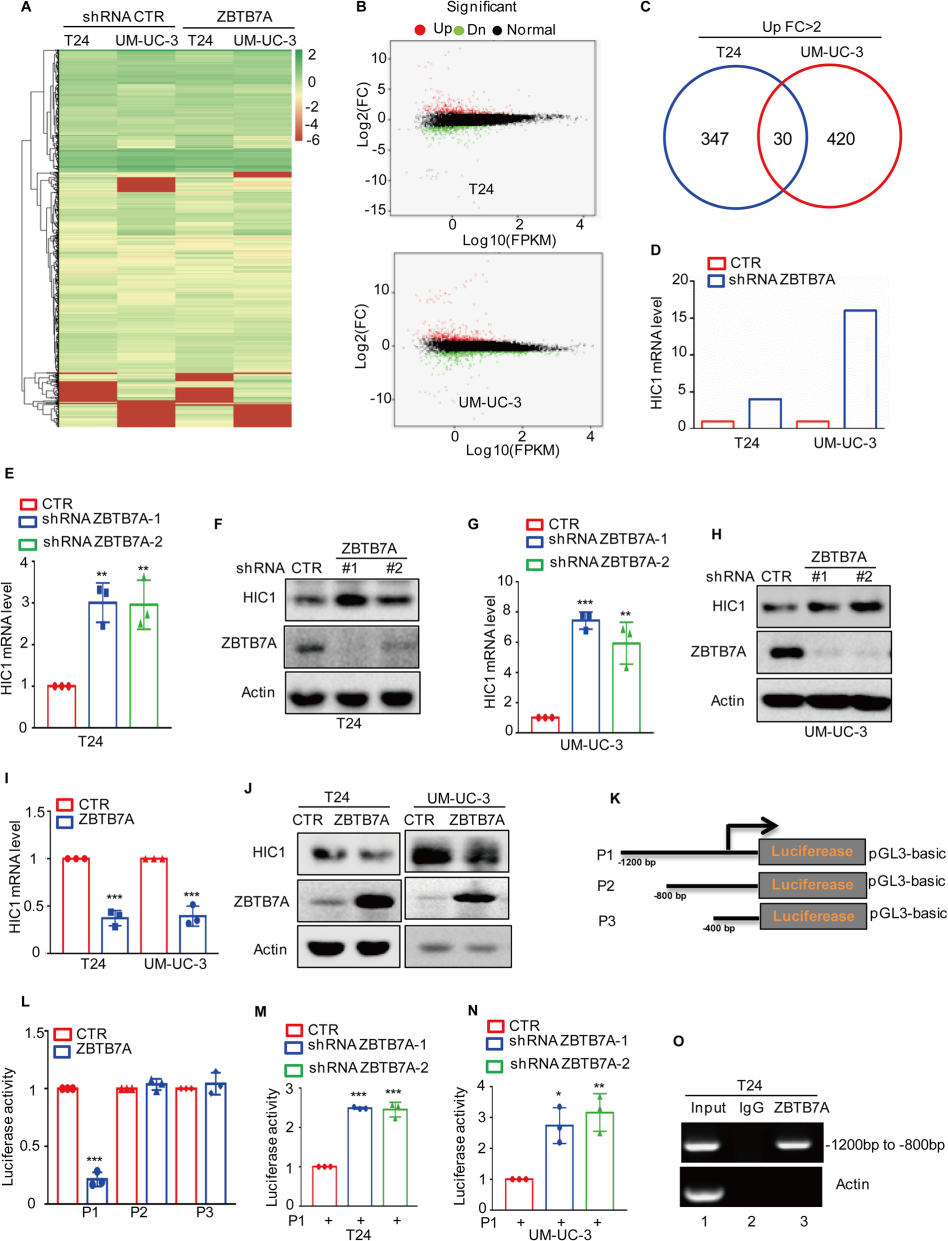
ZBTB7A regulates HIC1 levels. **A–B** T24 and UM-UC-3 cells with or without ZBTB7A knockdown were subjected to RNA sequencing analysis and illustrated as heatmap and volcano maps. **C** Venn diagram show overlapping genes in T24 and UM-UC-3 with or without ZBTB7A knockdown. **D** Changed folds of HIC1 mRNA were listed. **E–H** Expression levels of HIC1 were measured using qRT-PCR and Western blotting in T24 and UM-UC-3 cells with or without ZBTB7A knockdown. **I–J** Expression levels of HIC1 were detected using qRT-PCR and western blotting in T24 and UM-UC-3 cells with or without ZBTB7A overexpression. **K** Schematic illustration of pGL3-based reporter constructs used to assess the transcriptional activity of HIC1 using luciferase assay. **L** HIC1 promoters P1, P2 and P3 were transfected into 293 T cells, and then the luciferase activity was measured. **M–N** P1 was transfected into T24 and UM-UC-3 cells with or without ZBTB7A knockdown, and then luciferase activity was measured. **O** ChIP analysis showed the binding of ZBTB7A to HIC1 promoter in T24 cells using ZBTB7A antibody. An isotype-matched IgG was used as a negative control. All results represent three independent experiments and presented as the mean ± SD. *p < 0.05, **p < 0.01 and ***p < 0.001 compared with the control group

To elucidate the molecular mechanism underlying ZBTB7A's role in enhancing BC cell proliferation and migration, RNA sequencing was used to evaluate differentially expressed genes (DEGs) between ZBTB7A knockdown BC cells and control cells. Over 300 genes were upregulated in each cell line after ZBTB7A knockdown (Fig. 2A, B), and the Venn diagram demonstrates that 30 DEGs genes overlapped between T24 and UM-UC-3 cells (Fig. 2C).

Among these overlapping genes, the HIC1 was significantly upregulated in ZBTB7A knockdown cells (Fig. 2D), and its well-known tumor suppressor gene plays a critical role in various cancers [19]. One study has been shown, that HIC1 inhibits cell growth and migration via regulating YAP pathway in BC [20], thus we chose HIC1 for the further study. To replicate this finding, we used quantitative RT-PCR (qRT-PCR) and Western blotting to determine the expression levels of HIC1 in ZBTB7A knockdown cells. The results indicated that ZBTB7A knockdown upregulated HIC1 expression in BC cells (Fig. 2E–H), whereas overexpression of ZBTB7A suppressed HIC1 expression (Fig. 2I, J).

The downregulation effect of ZBTB7A on HIC1 expression in BC cells was assessed by cloning the promoter regions of HIC1 into pGL3-based luciferase reporter plasmids, which were labeled P1-P3 (Fig. 2K), and then transfecting these plasmids into 293 T cells with or without ZBTB7A overexpression. We discovered that ZBTB7A inhibited the luciferase activity of P1 but not of P2 or P3 (Fig. 2L), and thus P1 was transfected into T24 and UM-UC-3 cells with or without ZBTB7A knockdown. The results showed that ZBTB7A knockdown increased P1 luciferase activity, (Fig. 2M, N). Furthermore, chromatin immunoprecipitation (ChIP) analysis verified that the region (− 1200 to − 800 bp) was specifically present in anti-ZBTB7A immunoprecipitates from T24 cells (Fig. 2O), implying that the region (− 1200 to − 800 bp) constituted a key locus for ZBTB7A control of HIC1. The LINC00473 promoter was employed as a positive control because it was previously shown to be bound by ZBTB7A (Additional file 1: Fig. S1D) [12].

### ZBTB7A promotes BC cell growth and migration by reducing HIC1 expression

**Fig. 3:**
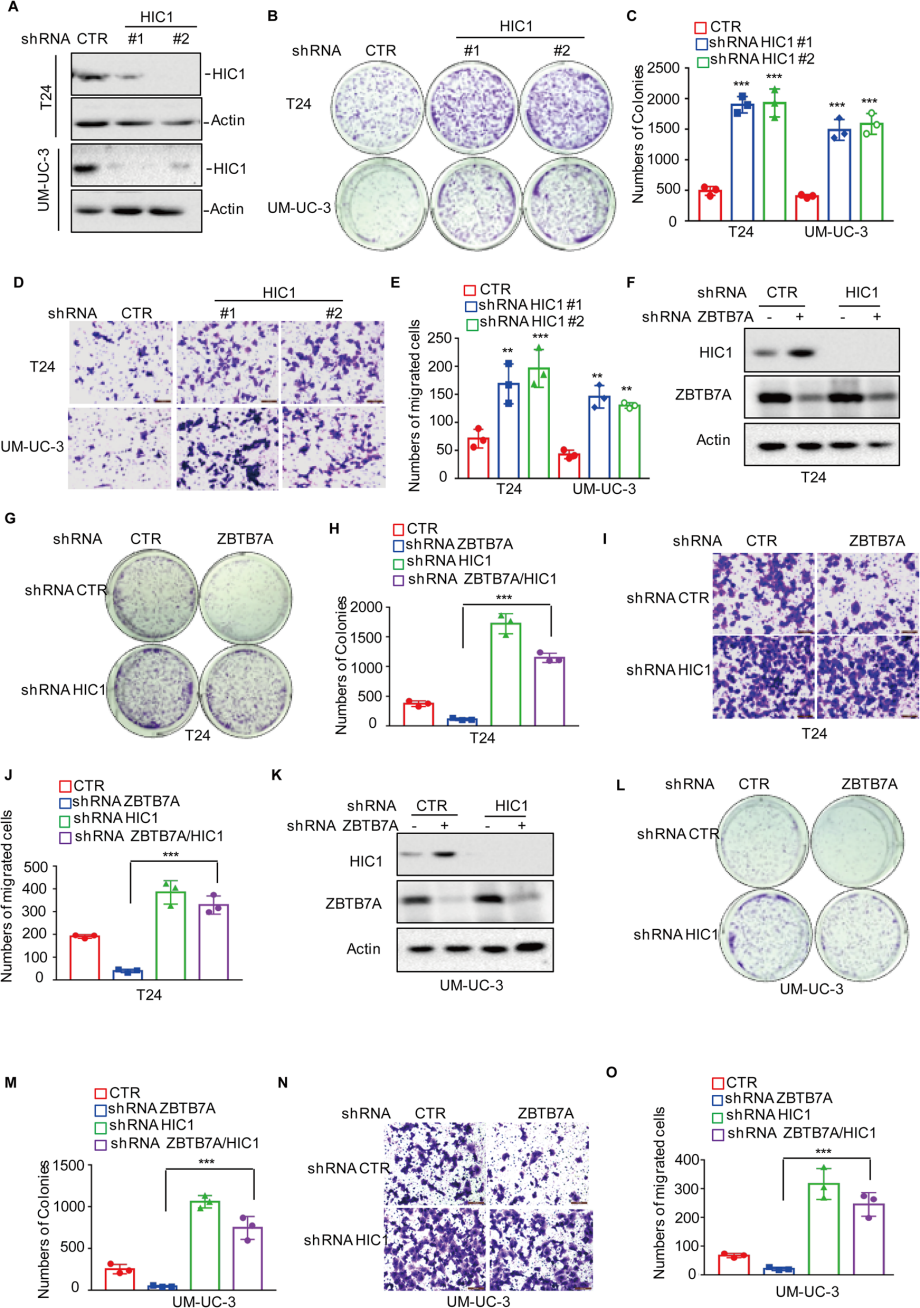
ZBTB7A promotes **B–C** tumorigenesis via regulating HIC1. **A** HIC1 was knocked down in T24 and UM-UC-3 cells. The expression levels of HIC1 were detected using Western blotting. **B**–**E** Cell growth and migration were assessed by colony formation and Transwell assays for knockdown HIC1 BC cells (T24 and UM-UC-3). **F** HIC1 was knocked down in T24 cells with or without ZBTB7A knockdown. The expression levels of HIC1 and ZBTB7A were analyzed using western blotting. **G**–**J** Cell growth and migration were assessed by colony formation and Transwell assays for knockdown HIC1 T24 cells with or without ZBTB7A knockdown. **K** HIC1 was knocked down in UM-UC-3 cells with or without ZBTB7A knockdown. The expression levels of HIC1 and ZBTB7A were analyzed using western blotting. **L**–**O** Cell growth and migration were assessed by colony formation and Transwell assays for knockdown HIC1 UM-UC-3 cells with or without ZBTB7A knockdown. All results represent three independent experiments and presented as the mean ± SD. *p < 0.05, **p < 0.01 and ***p < 0.001 compared with the control group in Fig. 3 C and E, compared with shRNA ZBTB7A group in Fig. 3**H**, **J**,**M** and **O**

To assess whether ZBTB7A promoted BC cellular proliferation by suppressing HIC1, HIC1 was first knocked down in BC cells with two independent shRNAs (Fig. 3A), and the effects of HIC1 on BC cell growth and migration were analyzed (Fig. 3B–E). Knockdown and low levels of HIC1 were shown to significantly increase BC cell growth and migration.

Subsequently, HIC1 expression was knocked down in T24 cells with or without ZBTB7A knockdown (Fig. 3F). The results revealed that HIC1 knockdown partly recovered cell growth and migration that was suppressed by ZBTB7A knockdown (Fig. 3G–J). Similar results were obtained in UM-UC-3 cells (Fig. 3K–O). Collectively, these findings indicate that ZBTB7A promoted BC growth and migration partly dependent on regulating HIC1 expression.

### Increased expression of ZBTB7A negatively correlated with HIC1 expression in BC tissues

**Fig. 4:**
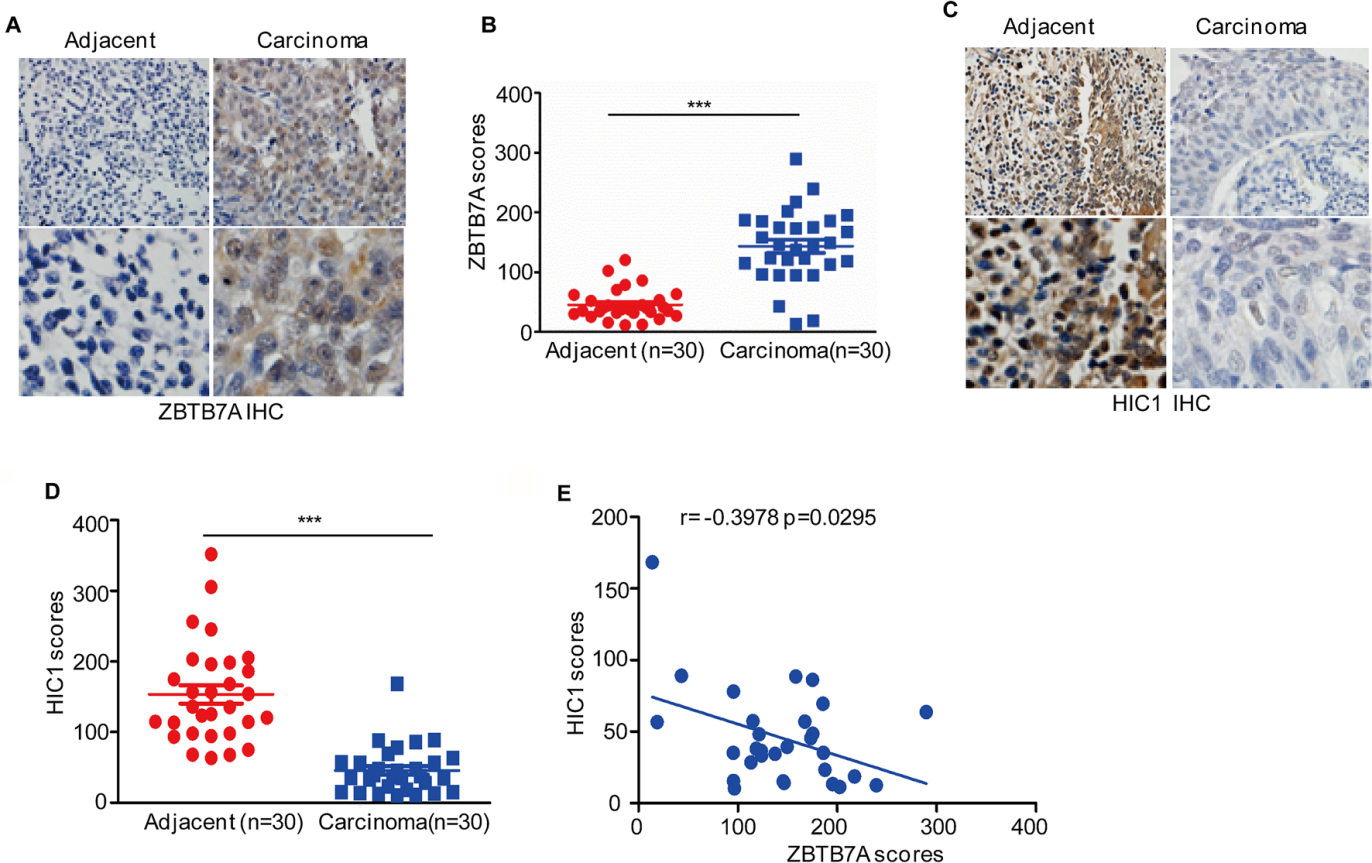
Increase of ZBTB7A in BC is associated with HIC1 protein expression. **A–D** Representative images from immunohistochemical staining of ZBTB7A and HIC1 in bladder cancer tissues (*n* = 30) and matched adjacent tissue (*n* = 30) respectively. Cancer tissues were compared with adjacent tissues using Wilcoxon or unpaired t-test. **E** Correlation between ZBTB7A and HIC1 protein levels was analyzed. Data were analyzed using Pearson correlation. *p < 0.05, **p < 0.01 and ***p < 0.001 compared with the adjacent tissue

To analyze the clinical importance of the ZBTB7A-HIC1 axis and determine their role in BC, we performed IHC staining to examine the expression of these proteins in serial sections of tissue microarrays containing BC tissues (n = 30) and adjacent tissues (n = 30). The IHC results showed that the expression levels of ZBTB7A were significantly upregulated in BC tissues compared with adjacent tissues, and HIC1 was significantly downregulated (Fig. 4A–D). Moreover, ZBTB7A exhibited a negative correlation with HIC1 in BC tissues (Fig. 4E).

### miR-144-3p suppresses ZBTB7A expression in BC cells

Increasing evidence suggests that miRNAs are significant transcriptionally independent regulators of ZBTB7A expression [11, 21–23]. In addition, miR-144-3p has been shown to be downregulated in BC [24]. As a result, we aim to find out whether miRNAs have a role in regulating ZBTB7A expression in BC cells.

**Fig. 5:**
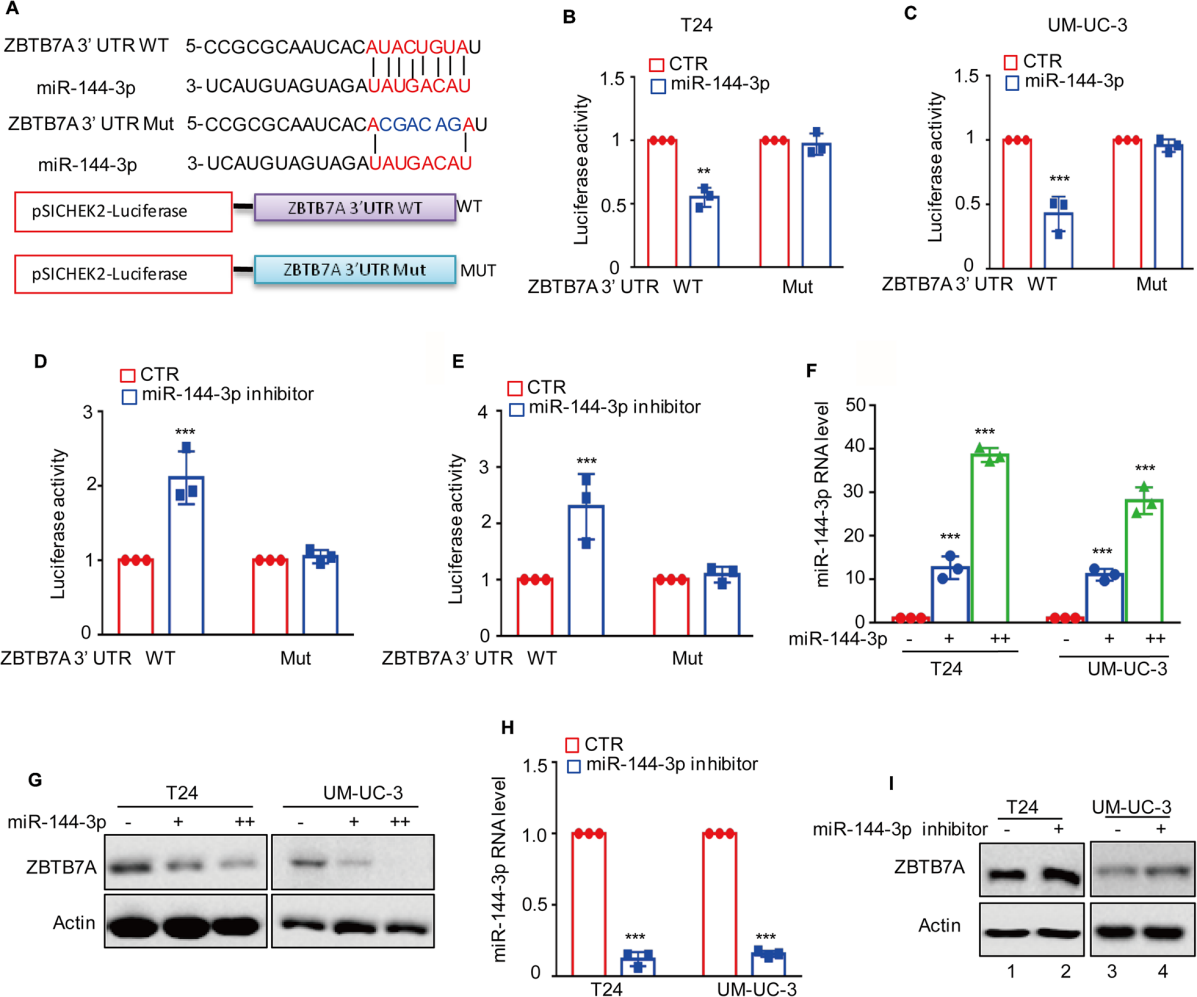
miR-144-3p regulates ZBTB7A expression. **A** Potential binding region of miR-144-3p on ZBTB7A was predicted by TargetScan. The sequences of wild-type or mutant ZBTB7A 3ʹUTR containing the putative of the miR-144-3p binding site were cloned into a pSICHECK2 vector, where blue indicates the mutated region. **B**, **C** Wild type (WT) and mutant (MUT) ZBTB7A 3ʹUTR were transfected into T24 and UM-UC-3 cells with or without miR-144-3p overexpression. The luciferase activity was measured. **D, E** WT and MUT ZBTB7A 3’UTR were transfected into T24 and UM-UC-3 cells with or without miR-144-3p inhibitors. The luciferase activity was measured. **F**, **G** Increasing amount of miR-144-3p was transfected into T24 and UM-UC-3 cells. The protein levels of ZBTB7A were analyzed with Western blotting. The expression levels of miR-144-3p were analyzed using qRT-PCR. **H**, **I** the miR-144-3p inhibitor was transfected into T24 and UM-UC-3 cells. After 36 h, the protein levels of ZBTB7A were analyzed with Western blotting. The expression levels of miR-144-3p were analyzed with qRT-PCR. All results represent three independent experiments and presented as the mean ± SD. *p < 0.05, **p < 0.01 and ***p < 0.001 compared with the control group

To investigate the relationship between miR-144-3p and ZBTB7A, the wild-type or mutant 3ʹUTR of ZBTB7A containing the putative of the miR-144-3p binding site were cloned into a dual-luciferase vector (Fig. 5A). Then, these plasmids were transfected into T24 and UM-UC-3 cells together with or without miR-144-3p and we found the luciferase activity of wild-type 3ʹUTR of ZBTB7A was decreased in miR-144-3p overexpressing cells (Fig. 5B, C). However, no decrease of the luciferase activity when the binding site was mutated. When we used the miR-144-3p inhibitor to block the miR-144-3p, we noticed that the luciferase activity of the wild-type ZBTB7A 3'UTR increased, whereas the mutant ZBTB7A 3'UTR was unaffected (Fig. 5D, E).

To further confirm these findings, the effect of miR-144-3p on the protein level of ZBTB7A in BC cells was assessed. miR-144-3p mimics and inhibitors were transfected into T24 and UM-UC-3 cells and the expression levels of miR-144-3p were detected using qRT-PCR (Fig. 5F), and protein levels of ZBTB7A were analyzed using Western blotting. The results showed that overexpression of miR-144-3p suppressed ZBTB7A expression in a dose-dependent manner (Fig. 5G). Conversely, inhibition of miR-144-3p elevated the protein levels of ZBTB7A (Fig. 5H, I). Taken together, these data indicate that ZBTB7A is a true target of miR-144-3p.

### miR-144-3p suppresses the malignancy of BC cells and upregulates HIC1 expression by targeting ZBTB7A

**Fig. 6:**
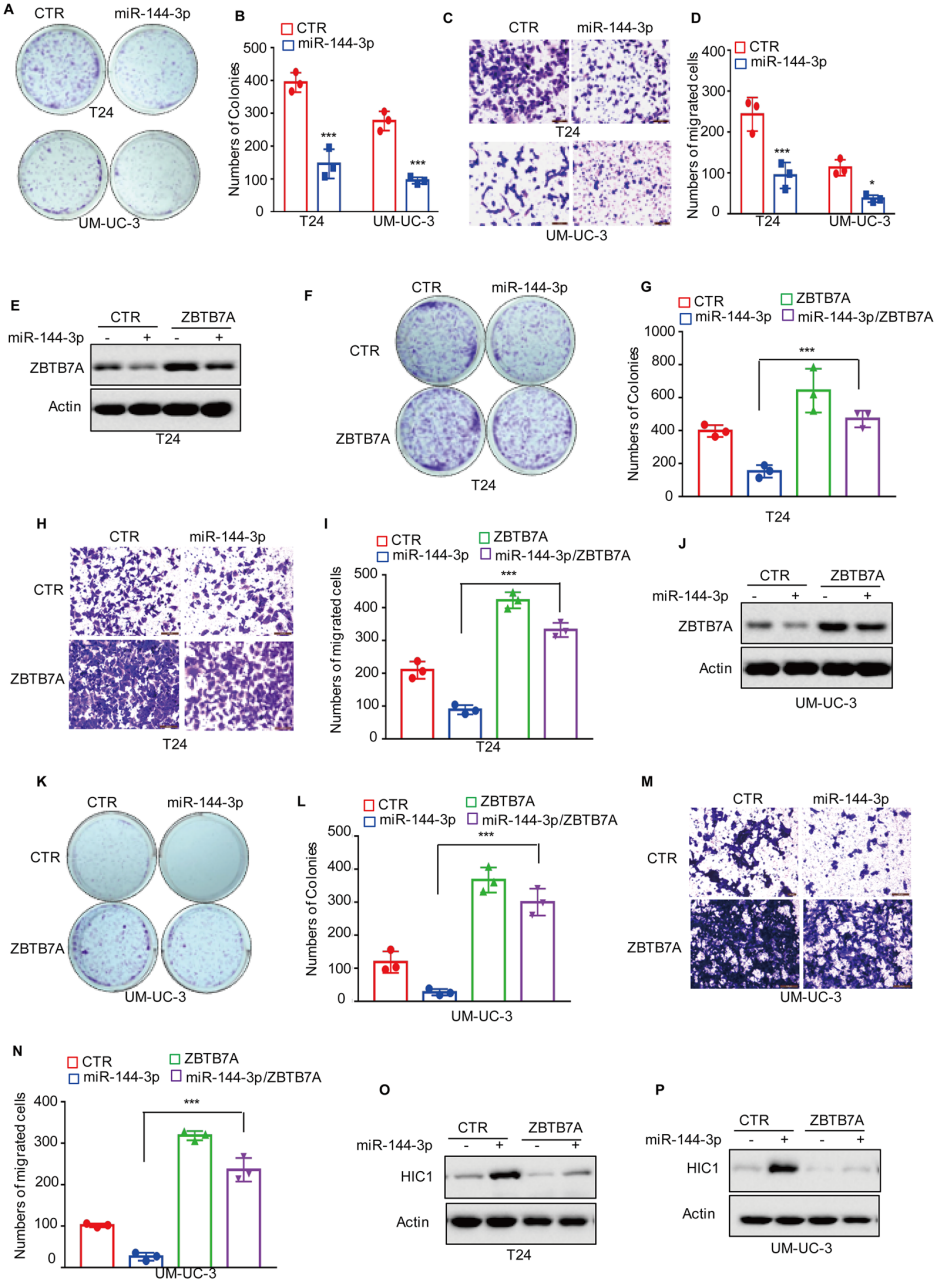
miR-144-3p suppresses the malignancy of BC cells and increases HIC1 expression via targeting ZBTB7A. **A, B** miR-144-3p was transfected into T24 and UM-UC-3 cells. After 7 days, Cell growth were assessed by colony formation. **C, D** miR-144-3p was transfected into T24 and UM-UC-3 cells. After 36 h, Cell migration was assessed by transwell assay. **E–G** miR-144-3p was transfected into T24 cells with or without ZBTB7A overexpression. After 7 days, the expression levels of ZBTB7A were detected by Western blotting and cell growth were assessed by colony formation. **H, I** miR-144-3p was transfected into T24 cells with or without ZBTB7A overexpression. After 36 h, Cell migration was assessed by transwell assay. **J, L** miR-144-3p was transfected into UM-UC-3 cells with or without ZBTB7A overexpression. After 7 days, the expression levels of ZBTB7A were detected by Western blotting and cell growth were assessed by colony formation. **M, N** miR-144-3p was transfected into UM-UC-3 cells with or without ZBTB7A overexpression. After 36 h, Cell migration was assessed by transwell assay. **O, P** miR-144-3p was transfected into T24 and UM-UC-3 cells with or without ZBTB7A overexpression. After 36 h, the expression levels of HIC1 were analyzed using Western blotting. All results represent three independent experiments and presented as the mean ± SD. *p < 0.05, **p < 0.01 and ***p < 0.001 compared with the control group in (**B** and **D)**, compared with the miR-144-3p group in (**G**, **I**, **L**, **N**)

**Fig. 7:**
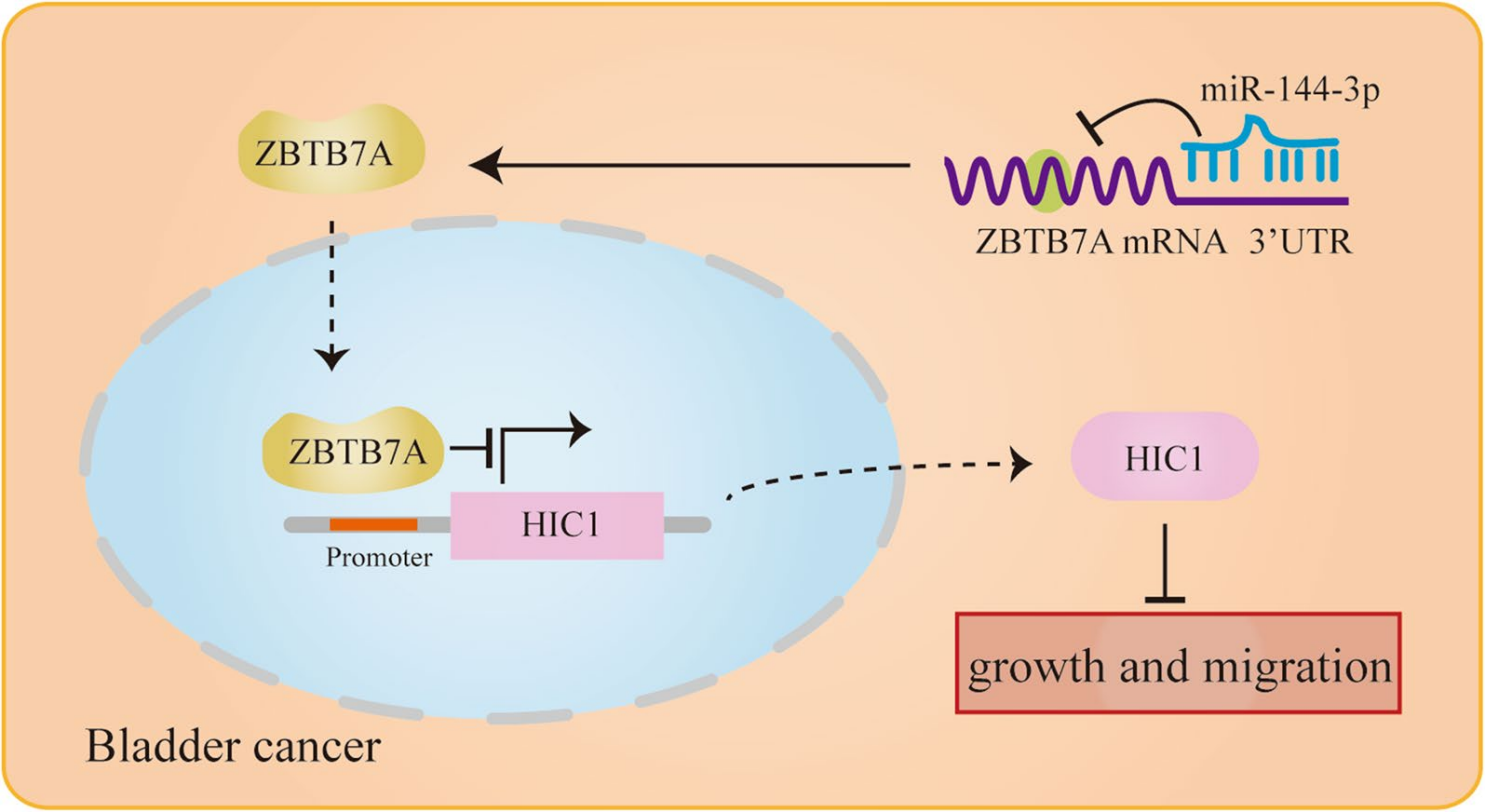
The schematic illustration of the miR-144-3p/ZBTB7A/HIC1 axis in BC

According to the above-mentioned findings, ZBTB7A can promote BC cell growth and migration by regulating HIC1 expression, we aim to explore whether miR-144-3p regulated the progression of BC by targeting the ZBTB7A-HIC1 axis. Therefore, the effects of miR-144-3p on cell proliferation and migration in BC were investigated. As shown in Fig. 6A–D, overexpression of miR-144-3p significantly inhibits cell growth and migration. Next miR-144-3p was transfected into BC cells with or without ZBTB7A overexpression and predicted expression levels of ZBTB7A and miR-144-3p were analyzed using Western blotting and qRT-PCR (Fig. 6E, J and Additional file 1: Fig. S1E, F). Furthermore, the alteration of cell proliferation and migration was assessed using colony formation and transwell assays. It was found that increased miR-144-3p expression significantly inhibited cell growth and migration; however, overexpression of ZBTB7A abolished the inhibitory effect of miR-144-3p (Fig. 6 F–I and K–N). Similarly, the increase of HIC1 by miR-144-3p was abolished by ZBTB7A overexpression (Fig. 6O, P). In summary, our findings demonstrated that miR-144-3p inhibited the malignancy of BC cells (Fig. 7) and increased HIC1 expression via targeting ZBTB7A.

## Discussion

There are studies in recent years investigated the role of ZBTB7A, which has been proved to play an oncogenic or tumor-suppressive role in multiple types of cancers. Although only a few research have identified the effects of ZBTB7A on BC epithelial-mesenchymal transition, the role and regulatory mechanism of ZBTB7A in BC are still limited. The current study found that ZBTB7A expression was significantly increased in BC tissues compared to normal bladder tissue. Increased expression of ZBTB7A promoted BC cell growth and migration by transcriptionally suppressing HIC1 expression. Additionally, ZBTB7A was a target of miR-144-3p, which decreased ZBTB7A expression in BC.

The function of ZBTB7A in the tumor is controversial and accumulating evidence indicates that ZBTB7A regulates several cell survival, differentiation and death signaling in diverse types of cancers [5, 25]. Previous studies showed that ZBTB7A suppressed tumorigenesis in melanoma and prostate cancer [13, 15]. ZBTB7A is essential for terminal erythroid differentiation while also blocking glycolysis, directly correlates with the observation that 2DG promotes erythrocytic differentiation of HSCs [26, 27]. However, ZBTB7A was originally known as a proto-oncoprotein due to its ability to suppress the transcription of tumor suppressor gene ARF [28]. ZBTB7A was increased in tumor tissues and promoted tumorigenesis in colorectal cancer, glioma and osteosarcoma [9, 11, 29]. ZBTB7A was only reported to regulate the epithelial-mesenchymal transition in BC [30, 31]. Our data indicated that ZBTB7A functioned as an oncogene and enhanced cell growth and migration via transcriptionally downregulating HIC1 expression. However, we found that depletion of HIC1 could not completely recover the decrease of cell growth and migration by ZBTB7A knockdown. These data indicated that other genes regulated by ZBTB7A may involve in promoting the BC cell growth and migration. Additionally, ZBTB7A expression was increased in BC tissues and was found to be inversely correlated with HIC1 expression.

HIC1 is an important tumor suppressor gene localized on human chromosome 17 p13.3 and closed to p53 [32]. HIC1 resides completely within a CpG island that is frequently hypermethylated in human tumors, including breast, prostate, and lung cancer. In bladder cancer, the promoter of HIC1 existed methylation statuses and the expression of HIC1 was downregulated in BC [19, 33, 34]. HIC1 was reported to inhibit cancer progression via targeting the Yap pathway in BC [20]. Similarly, our data showed that HIC1 expression was decreased in BC tissues and knockdown of HIC1 promoted cell growth and migration. Previous reports have suggested that promoter hypermethylation is the main reason for the decrease of HIC1 in tumor tissues. Our study found that ZBTB7A was a true transcription factor of HIC1, which suppressed HIC1 expression in BC cells.

The present study found that ZBTB7A was increased in BC tissues. However, the molecular mechanism underlying increased ZBTB7A in BC is still unknown. Previous reports showed that some miRNAs regulated ZBTB7A expression in cancers [22, 35]. For example, miR-100 suppressed ZBTB7A expression in gastric cancer [21], whereas miR-663a downregulated ZBTB7A expression in osteosarcoma [11], and MiR-372 inhibited ZBTB7A expression in oral carcinoma [23]. Our study revealed that miR-144-3p decreased ZBTB7A expression in bladder cancer. MiR-144-3p has been shown to suppress tumor development and metastasis in a variety of malignancies. MiR-144-3p, for example, inhibited gastric cancer growth and stemness by targeting GLI2 [36]. Another study found that inhibiting miR-144-3p accelerates the growth of non-small cell lung cancer by targeting CEP55 [37]. Similarly, by targeting ZEB1/2, MiR-144-3p suppressed colorectal cancer growth and metastasis [38]. Finally, MiR-144-3p inhibits cervical cancer growth by specifically targeting MAPK6 [39]. Our findings suggested that miR-144-3p inhibited BC cell proliferation and migration by targeting the ZBTB7A-HIC1 axis.

## Conclusions

In summary, this study indicates that ZBTB7A is an oncogenic driver in BC and the miR-144-3p-ZBTB7A-HIC1 axis plays a key role in BC progression.

## Supplementary Information

**Additional file 1** : **Figure S1**. **A**, **B** ZBTB7A was knocked down in T24 and UM-UC-3 cells. Cell death was assessed by flow cytometric analysis. **C** The expression of ZBTB7A in tumor xenografts were detected by Western blotting. **D** ChIP analysis showed the binding of ZBTB7A to LINC00473 promoter in T24 cells using ZBTB7A antibody. **E**, **F** miR-144-3p was transfected into T24 and UM-UC-3 cells with or without ZBTB7A overexpression. After 7 days, the expression levels of miR-143-3p were detected by qRT-PCR. All results represent three independent experiments and presented as the mean ± SD. *p < 0.05, **p < 0.01 and ***p < 0.001 compared with the control group.

## Data Availability

The data that support the findings of this study are available from the corresponding author upon reasonable request.

## Abbreviations

ZBTB7A: Zinc finger and BTB domain-containing 7A
BC: Bladder cancer
HIC1: Hypermethylated in cancer 1
POK: Pokemon
LRF: Lymphoma-related factor
FBI-1: Factor binding IST protein 1
TV: Tumor volume
DEGs: Differentially expressed genes
ChIP: Chromatin immunoprecipition
