# Supplementary figures and images for: ZBTB7A, a miR-144-3p targeted gene, accelerates bladder cancer progression via downregulating HIC1 expression
Source: Cancer Cell Int. 2022 May 2;22:179. doi: 10.1186/s12935-022-02596-w (PMC9063087; doi:10.1186/s12935-022-02596-w)

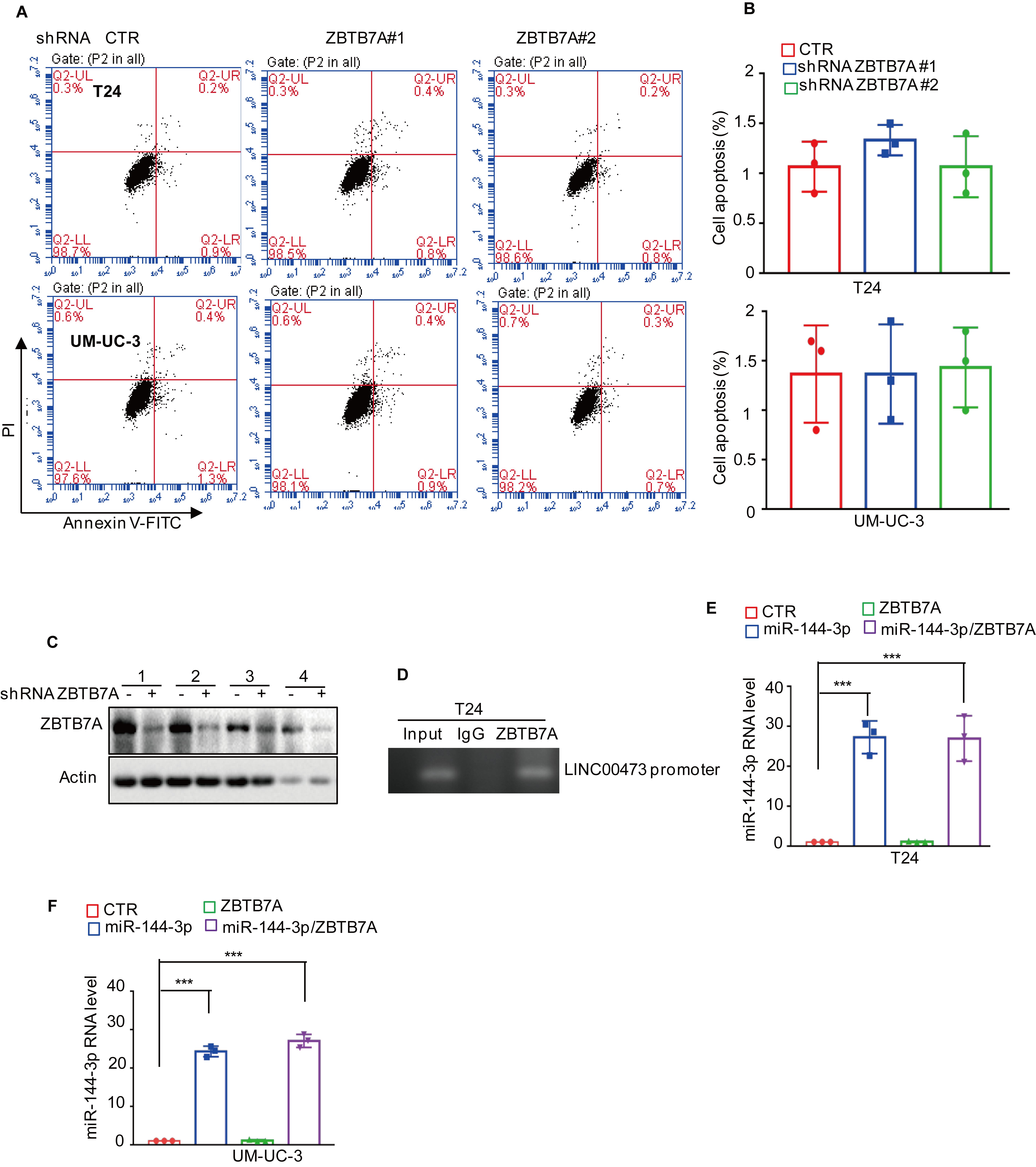

Supplement: Supplementary file 1 — Additional file 1 : Figure S1. A, B ZBTB7A was knocked down in T24 and UM-UC-3 cells. Cell death was assessed by flow cytometric analysis. C The expression of ZBTB7A in tumor xenografts were detected by Western blotting. D ChIP analysis showed the binding of ZBTB7A to LINC00473 promoter in T24 cells using ZBTB7A antibody. E, F miR-144-3p was transfected into T24 and UM-UC-3 cells with or without ZBTB7A overexpression. After 7 days, the expression levels of miR-143-3p were detected by qRT-PCR. All results represent three independent experiments and presented as the mean ± SD. *p < 0.05, **p < 0.01 and ***p < 0.001 compared with the control group. [file 12935_2022_2596_MOESM1_ESM.tif]
